# Traumatic axonal injury (TAI): definitions, pathophysiology and imaging—a narrative review

**DOI:** 10.1007/s00701-020-04594-1

**Published:** 2020-10-02

**Authors:** Gavin F. Bruggeman, Iain K. Haitsma, Clemens M. F. Dirven, Victor Volovici

**Affiliations:** Department of Neurosurgery, Erasmus MC University Medical Center, Rotterdam, The Netherlands

**Keywords:** Traumatic axonal injury, DAI, TAI, TBI, Pathophysiology

## Abstract

**Introduction:**

Traumatic axonal injury (TAI) is a condition defined as multiple, scattered, small hemorrhagic, and/or non-hemorrhagic lesions, alongside brain swelling, in a more confined white matter distribution on imaging studies, together with impaired axoplasmic transport, axonal swelling, and disconnection after traumatic brain injury (TBI). Ever since its description in the 1980s and the grading system by Adams et al., our understanding of the processes behind this entity has increased.

**Methods:**

We performed a scoping systematic, narrative review by interrogating Ovid MEDLINE, Embase, and Google Scholar on the pathophysiology, biomarkers, and diagnostic tools of TAI patients until July 2020.

**Results:**

We underline the misuse of the Adams classification on MRI without proper validation studies, and highlight the hiatus in the scientific literature and areas needing more research. In the past, the theory behind the pathophysiology relied on the inertial force exerted on the brain matter after severe TBI inducing a primary axotomy. This theory has now been partially abandoned in favor of a more refined theory involving biochemical processes such as protein cleavage and DNA breakdown, ultimately leading to an inflammation cascade and cell apoptosis, a process now described as secondary axotomy.

**Conclusion:**

The difference in TAI definitions makes the comparison of studies that report outcomes, treatments, and prognostic factors a daunting task. An even more difficult task is isolating the outcomes of isolated TAI from the outcomes of severe TBI in general. Targeted bench-to-bedside studies are required in order to uncover further pathways involved in the pathophysiology of TAI and, ideally, new treatments.

## Introduction

Traumatic axonal injury (TAI) is a condition characterize as multiple, scattered, small hemorrhagic, and/or non-hemorrhagic lesions, alongside brain swelling, in a more confined white matter distribution on imaging studies, together with impaired axoplasmic transport, axonal swelling, and disconnection with more than 3 such foci present on imaging studies according to the National Institutes of Health Common Data Elements [1]. In the past, DAI (diffuse axonal injury) was defined as prolonged (> 6 h) loss of consciousness (LOC), without a visible mass lesion.

TAI was first described mid-twentieth century, as diffuse microscopic pathological changes to the brain tissue [2]. These lesions were believed to be the direct result of mechanical impact on brain tissue after trauma. This belief later relied on performing experimental observations using gelatine models of the human brain, where shear and tear was found after applying rotational force of relative intensity to those in a traumatic incident [3, 4].

At the start of the 1980s, the term DAI (diffuse axonal idiopathic injury, nowadays called TAI) was introduced in consecutive studies by Adams and Gennarelli, among others [5, 6]. The term DAI implicates that there is diffuse topographic distribution of traumatic findings. However, studies showed that the distribution of traumatic lesions in DAI has a predisposition for white matter tracts in the midline of the brain, including the corpus callosum, internal capsule, cerebral peduncles, brainstem, and the grey-white junction of the cerebral cortex [5, 7]. These findings are probably related to the type of post-mortem examination done in the study with higher prevalence of central traumatic lesions after high energy trauma. Nowadays, the term TAI, or traumatic axonal injury, is more accurate than the term DAI.

TAI is thought to be caused by a variety of traumatic mechanisms involving fast acceleration and/or deceleration, including motor vehicle accidents, falls from height, and blunt assault [6]. Adams et al. suggested a grading system for TAI, dividing TAI into three different subgroups (Table 1) [5]. In clinical practice, this grading has been applied clinically using MRI susceptibility-weighted (SW) imaging, but no thorough external validation studies have been carried out to confirm proper clinical use of the MRI grading.

**Table 1:** Grading of TAI, according to the histopathologic study of Adams et al. (3). Even though this grading appears to be 1:1 translatable to MRI findings, there is no study that validates histopathologic findings and MRI findings

Grade	Definition
Grade 1	Evidence of axonal damage in the white matter of the cerebral hemispheres including the corpus callosum, in the brain stem and, occasionally, in the cerebellum: this damage can only be identified microscopically.
Grade 2	Focal lesion in the corpus callosum in addition to diffuse axonal damage: the focal lesion was identifiable only microscopically. Can be said to be severe if the focal lesions are apparent macroscopically.
Grade 3	Focal lesions both in the corpus callosum and the dorsolateral quadrant of the rostral brain stem: the focal lesion was identifiable only microscopically. Can be said to be severe if the focal lesions are apparent macroscopically.

## Materials and methods

We performed a search from the first available records of each specific database to July 2020 using MEDLINE, Embase, Web of Science, and Google Scholar with the terms “diffuse axonal injury,” “traumatic axonal injury,” and “axonal injury.” The references of all included articles were also cross-checked for missed articles. Both clinical and basic science papers were included, describing the pathophysiology, diagnostic studies, and TAI-targeted treatments. Both pediatric and adult studies were included.

Two independent reviewers sifted through the title/abstracts and decided on the full-text inclusions. These were reviewed in the second sift phase and conflicts were resolved by consulting a third reviewer. A total of 160 articles were included.

We designed a scoping, narrative review with the objective to synthetize available information and identify the knowledge hiatus in the literature. A preliminary orientating search showed great heterogeneity in study methods and research type. Therefore, we did not expect enough comparable data and we also did not expect it to be of sufficient quality in order to perform a meta-analysis, so this was not defined in our study protocol. Therefore, we did not publish a protocol on Prospero, we did not plan any pooled analysis or sensitivity analysis, but we did perform a risk of bias analysis for most included papers.

### Pathophysiology

The pathophysiology of TAI is complex and lacking a unifying theory. The assumption that TAI is primarily and solely caused by direct mechanical force has been abandoned. Besides the primary damage, there is secondary damage caused by chemical alterations and changes in neuronal metabolism.

#### Trauma mechanism

There are two major mechanisms involved in head trauma: direct impact and accelerative and decelerative (a/d) forces [8]. Sudden head movement produces a force vector inside the intracranial cavity, resulting in shearing and strain injury. Shear and tear of axonal fibers can cause axonal damage, resulting in TAI [6].

The duration of the a/d forces is decisive for the type of injury. Slower A/d forces with a relatively long duration (20–25 ms) will mainly cause TAI, whereas shorter duration of a/d forces will likely cause acute subdural hematoma (ASDH) through shearing of bridging veins [6, 9]. A/d forces in the coronal plane are primarily associated with the occurrence of TAI [6, 10, 11]. The combination of translational and angular acceleration [12], but also rotational acceleration [13] has also been suggested as the most prominent cause of TAI.

The fact that TAI can be caused by traumas with relatively low rates of acceleration is of importance in forensic pathology. Deadly TAI can occur even if the initial impact force is not strong enough to cause fracturing of the skull or evident macroscopic pathology to the brain [10, 14].

#### Primary axotomy

The initial hypothesized pathophysiological mechanism was that pure mechanical stretch due to traumatic acceleration or deceleration alone led to direct tearing of axonal fibers. Subsequently to this tearing, the damaged axonal fibers would retract and form retraction bulbs, which are visible on pathological examination. This process of direct tearing is called primary axotomy [15, 16]. Gliding hemorrhages visible on neuroimaging right after trauma suggest that primary axotomy can occur directly after impact. Complete primary axotomy is nowadays considered a rare form of damage that occurs in cases of massive, widespread axonal damage [8, 17, 18]. Primary axotomy may also cause a decreased level of consciousness through widespread CNS damage including the thalami and the reticular substance of the brainstem.

#### Secondary axotomy: molecular basis

**Fig. 1:**
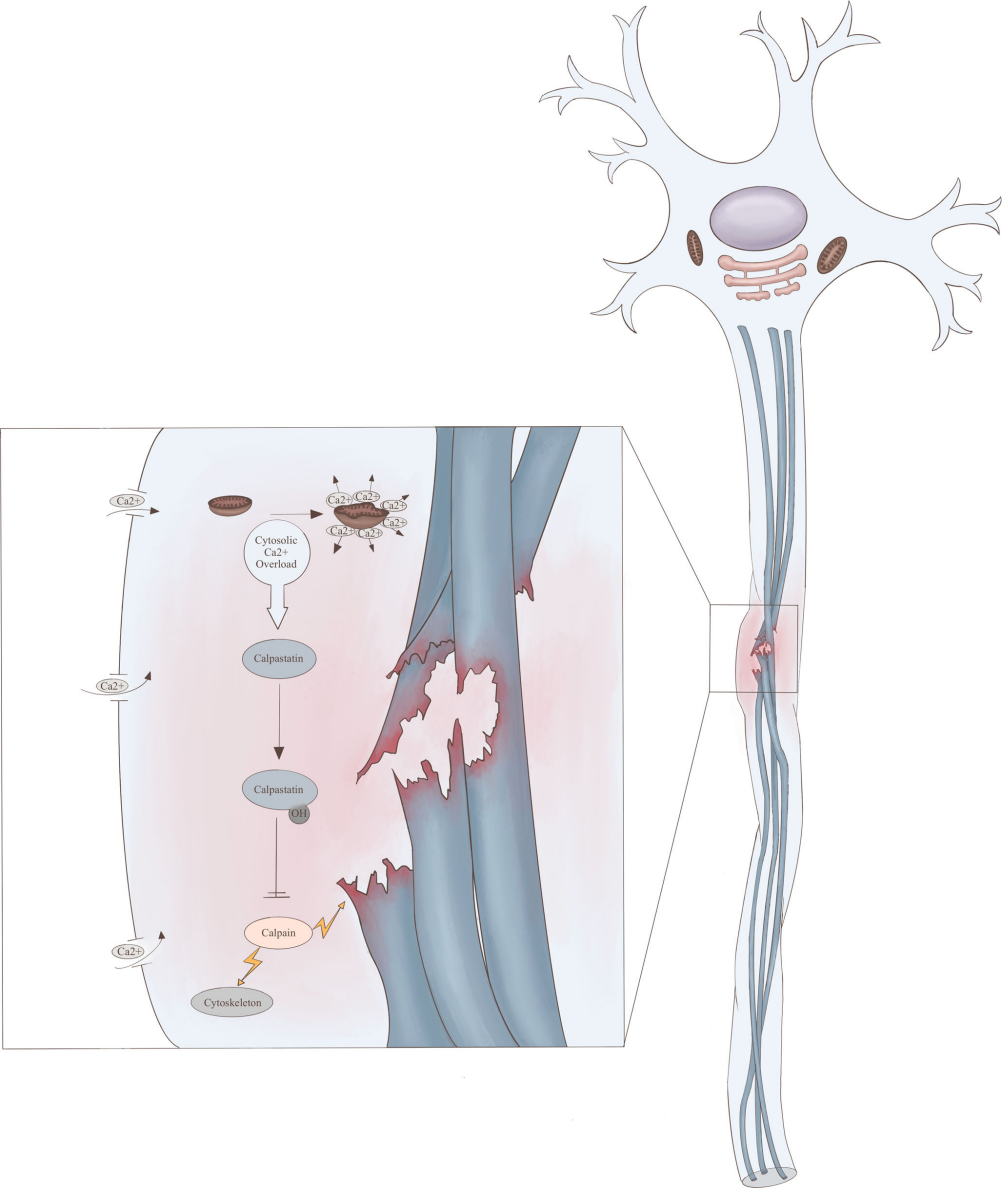
Primary and secondary axotomy pathophysiological processes (left-hand inlay), courtesy of M. W. T. van Bilsen, MD. The figure illustrates an axon upon which shear and rotational forces act. The microtubules (blue) become progressively stiffer and eventually break, leading to a disruption of the axonal transport of molecules. Calcium accumulates in the cell, both through the mechanical opening of calcium channels, as well as through the disruption of mitochondria. Through hydrolyzation of calpastatin (which normally inhibits calpain), calpain is activated and it in turn hydrolyzes the cytoskeleton and microtubules. This cascade leads to apoptosis and axon disconnection

If the inertial forces are of low intensity and do not cause complete primary axotomy, they can still be strong enough to cause partial damage to the axon, triggering a molecular pathway resulting in what is nowadays called secondary axotomy [19, 20]. Secondary axotomy is an inflammatory and apoptotic event (Fig. 1), which may create a potential window of opportunity for therapeutic treatment [21]. This window will probably only be a few hours. While the primary axotomy is a true mechanical event, caused by shearing forces, secondary axotomy can be seen as an apoptotic/neurodegenerative event which result in secondary loss of axons after trauma. Secondary axotomy can also be seen as a continuation of primary axotomy, where the initial structural damage caused by the traumatic forces forms the base for the entire molecular cascade, instead of as a separate entity.

The key element in secondary axotomy is axonal microtubules (MT). MT are subcellular structural cytoskeletal elements composed of long, stiff polymers of αβ-tubulin. MT are structurally and functionally important components of the eukaryotic cell cytoskeleton, which can serve as tracks for intracellular trafficking and energy-dependent directional intracellular transport [22]. MT are viscoelastic. With rapid stretch, the MT become the stiffest portion of the axon, making them viable to breaking (Fig. 1). MT breakage interrupts the axonal transport, leading to accumulation of transport proteins such as β-APP. The accumulation of these proteins causes axonal swelling, which can lead to disconnection of the axon from the neuron body if the process evolves severely enough. The primary mechanical breakage of MT can be followed by secondary depolymerisation of the remaining MT, disrupting axonal transport even more [23–26]. Mechanical damage to axonal microtubules itself can also be considered as incomplete primary axotomy.

Another major player in the secondary axotomy is the increased concentration of intracellular calcium ions [27]. Multiple mechanisms lead to increase in intracellular calcium, including activity of calcium-ATPase, the release of intra-axonal calcium storage, and the mechanical dysregulation of voltage-gated sodium channels [28–31].

The increased intracellular calcium concentration causes multiple intracellular processes leading to cell death. The cysteine and caspase-3 protein pathways become activated [32]. Hydrolyzation of calpastatin, a calpain inhibitor, leads to accumulation of calpain, which subsequently hydrolyses multiple structural and functional proteins within 1 to several hours after injury [27, 33–36]. Calpain-induced hydrolyzation of the sodium channel inactivation gate leads to progressively increasing intracellular calcium concentrations and calcium-associated damage [37]. Hydrolyzation of spectrin leads to severe damage to the cytoskeletal network. Microtubules and neurofilaments also get hydrolysed and thereby damaged. As a result of cytoskeletal degradation, axons get instable and disconnect [27, 38–50].

Calpain also has a role in ankyrin proteolysis. This may lead to distortion of the arrangement of sodium channels on the nodes of Ranvier. This pathway could contribute to instability of the axonal membrane [51].

Increased intracellular levels of calcium alter the permeability of mitochondrial membrane through the activation of calcineurin [52]. Activation of oxygen radicals also contributes to changes in mitochondrial membrane permeability. Calcium overload further causes swelling and rupture of the mitochondria. This influences homeostasis, energy metabolisms, and the release of caspases. Caspase hydrolyses proteins in severely damaged axons, resulting in caspase-mediated programmed cell death [53–56]. Mitochondrial failure leads to the inability to produce high energy phosphates, which are needed for the support of axonal functions and structure [56].

This concerted action of MT stiffness on the one hand and calcium overload with apoptosis and cell death are what ultimately lead to neuron dysfunction and widespread loss of connectivity. Secondary axotomy is nowadays accepted as the mechanism of neuronal dysfunction after TAI but the exact timing of events and the targeted treatment remain elusive.

Currently, the role played by myelin and glial cells in the process of secondary axotomy constitutes an important focus of research. In the central nervous system, wrapping of oligodendrocytes plasma membrane extensions forms the myelin sheath around the axons [57]. Myelin is thought to play a role in maintaining axon stability and integrity [58–60]. Animal studies reveal that there is significant loss of myelin in the corpus callosum and the brain stem after TAI. The vulnerability of oligodendrocytes and the demyelination that follows after oligodendrocyte apoptosis may contribute to axonal damage in the brainstem. Myelin and oligodendrocytes, as well as their interplay, might form a future therapeutic target for the treatment of TAI [61].

#### TAI, coma, and loss of consciousness

In the past, DAI was defined (due to a lack of more advanced imaging studies and limited understanding of pathophysiological mechanisms) by prolonged loss of consciousness (LOC) (> 6 h), in the absence of a visible mass lesion [62, 63]. Recently, isolated TAI as the sole etiology of coma has been called into question [64, 65].

Biomechanically, the plane of the brainstem relative to the rotational force might be involved in the occurrence and duration of LOC. Whereas rotation of the brainstem on its transverse axis was associated with coma, equal rotation on the vertical axis was not [56, 66]. LOC occurs thus even when small rotational forces act transversally to the brainstem [67].

The topographic distribution of axonal damage is also important for the occurrence of LOC [56]. Duration of coma has been linked directly to the severity of TAI in the brainstem, but more extensive bilateral thalamic damage or the massive, diffuse hemispheric damage likely also play a role [66]. Despite having an essential role, the brain stem is often not the only cause of coma in TAI patients.

### Diagnostics

#### Histopathological findings

Histopathology only plays a role in post-mortem diagnostics. It is not used in a clinical setting. The traditional histological findings in TAI are large axonal dilations caused by complete axotomy, referred to as retraction bulbs or axonal bulbs [2, 5, 23, 68–75]. Axonal varicosities are another histological finding. These are dilations along the length of the axon, visible within several hours after trauma, the result of processes described above under secondary axotomy due to axonal transport impairment and protein accumulation [19, 25, 26, 71].

Traditionally, hematoxylin and eosin (HE), and several silver stains were most widely used to detectpathological changes. Nowadays, immunohistochemical staining is more widely used. Accumulation of beta-amyloid precursor protein (β-APP) is a sensitive marker for diagnosis of TAI [76–80]. Accumulation of β-APP is visible within 2 h after trauma and shows more extensive injury than HE or silver staining. Because of these issues, β-APP immunostaining is the golden standard for pathological diagnosis of TAI [81–83]. It must be stated that the characteristic histological observations found appear after distinct times post-trauma and depend on the survival times of the individual affected (before the brain was harvested for pathological analysis).

#### Imaging studies

CT is capable of identifying large TAI-related hemorrhage, but non-hemorrhagic lesions and small TAI hemorrhage are virtually impossible toidentify using CT [84]. The slice thickness of a conventional trauma head CT is about 5–10 mm. Since TAI lesions may fall under this detection margin, they can be easily missed using conventional CT.

Conventional MRI (cMRI) has a higher sensitivity in demonstrating lesions in the brainstem and the deep white matter, making it more sensitive for identifying axonal injury compared to CT [85–88].

The MRI gradient echo sequence (GRE) is able to detect heme and heme breakdown products, making it a suitable method for discovering small hemorrhagic lesions [89].

Susceptibility weighted imaging (SWI) as a variant sequence of GRE imaging should be considered the “gold standard” for identifying TAI lesions. It has a higher sensitivity for hemorrhage than GRE , which makes it more useful for early diagnosis of TAI [84, 90]. However, association with outcomes has not yet been demonstrated and SWI might overestimate the size of a lesion due to its high sensitivity to heme products (Fig. 3).

Diffusion-weighted imaging (DWI) can accurately examine non-hemorrhagic lesions [84]. High signal DWI can be used in patients with early stage TAI [91]. Lesions found represent cellular swelling and cytotoxic oedema [92]. DWI may aid in predicting clinical outcome after TAI [93]. DWI is more capable of determining the severity of the injury and estimating the long-term prognosis than MRI techniques [94].

Diffusion tensor imaging (DTI) is an improved form of DWI. It can be used to evaluate nerve alignment, white matter microstructure and the morphology around nerve fibers [84]. Within the first 24 h after trauma, DTI can detect white matter regions with reduced anisotropy, making it an adequate technique for detecting TAI [95]. Anomalies found with DTI seem to be associated with both acute GCS and modified Rankin scores (mRS) at discharge [96]. Animal studies comparing DTI images and histological examination showed that DTI is highly sensitive for axonal injury and likely has a high negative predictive value [97, 98].

The direct correlation of histopathologic findings with MRI findings has not been validated in large human studies, which makes the interpretation of the TAI grade as proposed by Adams and colleagues [5] impossible based on imaging studies. Caution needs to be used when employing this tactic in a clinical setting, despite the practice being widespread.

The longer scanning time and inability to closely monitor patients undergoing MRI makes this technique less relevant in the acute phase after trauma, save for recent research endeavors. Many patients are not stabile enough to withstand the long scanning time used to make MRI images. CT-scanning is more widely used in the acute phase, due to its shorter scanning time. MRI scanning should be performed as soon as the condition of the patient allows it, so the full extent of trauma can be mapped and white matter volume prospectively followed-up.

#### Biochemical markers

Several potential biochemical markers have been investigated in TAI. Most of these are being investigated in the broader sense of their evolution post-severe TBI. C-tau, the polymerized version of the microtubule-related protein Tau, might be one of the most useful biomarkers for diagnosing TAI [99]. The detection level of C-tau in the cerebrospinal fluid (CSF) appears to be related to the severity of TAI in the clinical setting [45, 100]. The protein tau on itself is also a potential biomarker. Significantly higher serum levels of tau have been found in patients with TAI compared to patients without TAI; however, tau levels were not a predictor for unfavorable outcome in TAI patients [101].

Neurofilamental heavy chains (NF-H) are an emerging marker for diagnosing TAI, together with light chains (NF-L), which in turn appear to be a highly sensitive and specific marker for TAI [102, 103].

The biomarkers S-100B and neuron-specific enolase (NSE) are also used both in research and clinically. Both can be detected at increased levels in serum and CSF after TBI [104–114]. The serum biomarker levels seem to be associated with the cumulative size and number or contusion detected by CT-imaging [115]. S-100B correlates more with the severity of TBI [116]. Normal levels of S-100B after trauma might accurately exclude brain injury, as is the practice in northern countries [117].

Besides being the histological golden standard, β-APP is also of interest as a biochemical marker. Very basic research into β-APP derivatives in blood and in cerebrospinal fluid leads to the suggestion that 𝛽-APP derivatives in CSF could be an indicator of axonal damage [118].

Plasma levels of high-density lipoprotein cholesterol (HDL-C) are an emerging diagnostic biomarker to diagnose for TAI. In a retrospective study, HDL-C levels within 1 week after trauma of TAI patients were significantly lower than in the non-TAI control group. HDL-C was identified as an independent predictor for TAI after multivariable analysis [119].

Several studies in the field of proteomics have been conducted, generating long lists of potential biomarker proteins [120, 121]. Proteomics might be a promising field of research to find suitable biomarkers in the future.

A wealth of biomarker research is being conducted at this moment. Through better characterization of the disease phenotype, hope exists for potential future personalized treatment.

### Secondary axotomy-targeted treatment

Treatment of TAI mainly consists of treating underlying problems, the focal and systemic injuries. Currently, there is no TAI-specific treatment available. Treatment is performed in comformity with the “Guidelines for the Management of Severe Traumatic Brain Injury” [122].

**Fig. 2:**
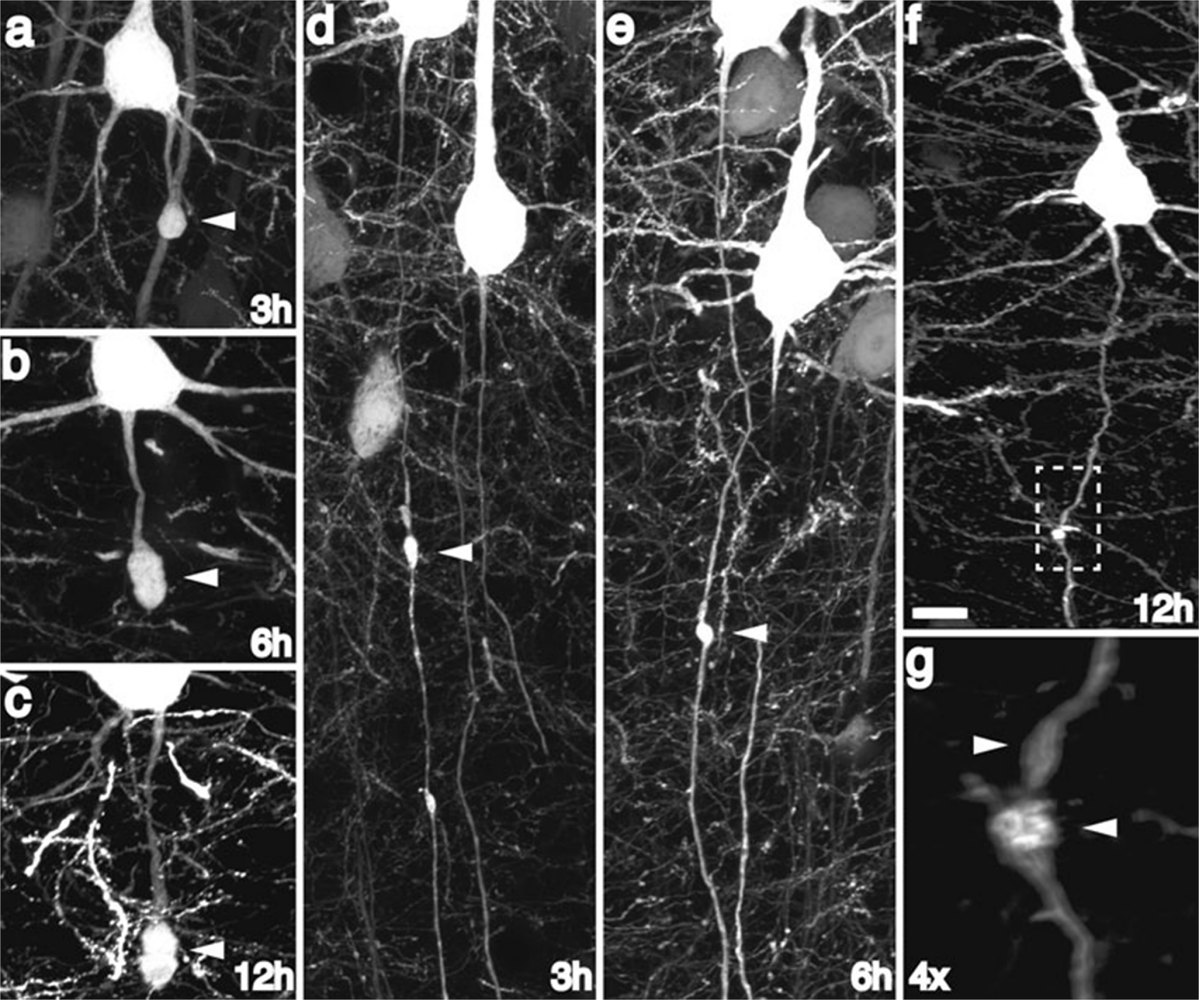
Reproduced with permission. YFP+ axonal swellings progressively enlarge and disconnect over time post-injury. Consistent with progressive axonal change, YFP+ swellings continue to enlarge over the next 12 h post-injury (**a**–**c**). Though the majority of swellings progress to disconnection over this 12-h time span, occasional swellings still maintaining axonal continuity could be found at later time points following injury (**d**–**g**), underscoring a temporally heterogeneous evolution of YFP+ swellings over time. Scale 10 μm. Reprinted by permission from Springer Nature: Acta Neuropathol Mild traumatic brain injury in the mouse induces axotomy primarily within the axon initial segment, Greer et al, Copyright 2013

However, a lot of research aims to identify possible treatment options based on the molecular theory explained above. Two main treatment types can be identified. The first type are treatments that focus on the prevention of secondary axotomy. Since secondary axotomy is an inflammatory and apoptotic event (Fig. 2), there may be a potential window of opportunity for therapeutic treatment [21]. The second type aims to enhancing neuronal regeneration after damage has already occurred.

#### Treatment preventing secondary axotomy

Multiple agents have been studied that influence calcium hemostasis. Administration of the Ca-channel blocker nimodipine could play a role in preventing or minimizing secondary damage of the axon. Preclinical studies showed a decreased expression of 𝛽-APP, suppressed activity of calcineurin (CaN), and lessened ultrastructural axonal damage [52]. Ciclosporin A (CsA) also inhibits CaN. By preventing a rise in mitchondrial membrane permeability, CsA abates swelling and disruption of mitochondria and thereby mitigates axonal damage [27, 38, 123, 124]. It also antagonizes calcium-mediated cytoskeletal disruption, and thereby secondary axotomy [27, 38, 125].

The calpain inhibitors MDL28170, 5b, AK295, and SJA-6017 seem to have a protective value; however, this protective effect has been disputed [126–130].

FK506 (also known as tacrolimus) seems to be less toxic than CsA [131]. It attenuates CaN, without a mitochondrial effect, even though this has been disputed in recent studies. Despite this, FK506 provides massive axonal protection and decreased axonal injury [132–134]. By reducing progressive damage to the cytoskeleton, FK506 also reduces secondary axotomy [131].

In a more recent study, the drugs SN-6 and dynasore have been proven to both reduce the extend of stretch injury-induced swelling of axonal varicosities and mitochondrial degradation. It also seems to protect against oxidative damage. SN-6 inhibits the the sodium calcium exchanger1 (NCX1), whereas dynasore inhibits several GTPase proteins (Drp1, dynamin1 and 2) which regulate mitochondrial cleavage [135].

Microtubules form another target for therapy. Taxol-like drugs (taxanes) are microtubule stabilizing drugs. Paclitaxel (Taxol) has possible effect on limiting axonal degeneration [26, 136, 137]. In vitro paclitaxel effectuated secondary depolymerization of microtubules, reducing axonal swelling and degeneration [26]. Since Paclitaxel cannot easily pass the blood-brain barrier, high IV doses are required leading to severe side-effects, such as peripheral neuropathy [138]. The microtubule stabilizing drug Epothilone D is more capable of passing the blood-brain barrier. It is shown to be beneficial in spinal cord injury, but there is no proof of its benefit in brain injury [139, 140].

Combination of hypothermia with drugs like FK506 or CsA during the rewarming phase might give additional protection [134, 141, 142]. This is either caused by a synergistic effect or because hypothermia reduces the progression of TAI, prolonging the window of opportunity for drug treatment [142–145].

Erythropoietin (EPO) has been shown improve both motor deficit and cognitive functions in an animal model, and was able to preserve axons in the injured area [146]. However, EPO can increase the calcium influx [147]. This can promote TAI progression in the early phase, so its applicability remains limited [148]. A recent RCT showed no improvement of neurological outcome 6 months after TBI after administration of erythropoietin [149, 150].

The neuroprotective properties of progesterone have also been studied. Results are conflicting. Studies show that progesterone might yield better learning, memory, cognitive, and motor functions and reduced axonal damage [151, 152], and improved neurological outcome on the long term [153]. However, a large meta-analysis showed no effect of progesterone in reducing mortality or unfavorable outcomes [154].

A recent study found altered expression of α7nAChR, a nicotine receptor, in a rat TAI model. They found that administration of a selective α7nAChR agonist yielded better learning and memory performance as compared to a α7nAChR antagonist [155].

#### Treatment enhancing neuronal regeneration

Stem cell therapy is studied not only in the field of TBI, but also in the field of ischemic brain damage. The therapeutic value of stem cells is traditionally believed to be based on two principles. There is thought to be direct cell replacement by stem cells. On the other hand, stem cells are thought to release neurotrophic factors, enhancing neuronal regeneration. A newer idea is that of bio-bridging. The idea behind this theory is that the stem cells form a connection between the areas on both sides of the neuronal damage. This so-called bio-bridge can form a pathway that promotes both proliferation and migration of native stem cells [156].

In the context of TAI, implantation of embryonal stem cells (ESC), neuronal derived stem cells (NSC), and bone-marrow-derived stem cells (BMSC) has been researched. Implantation of embryonal stem cells (ESC) after TAI may yield improvement in functional outcome [157]. The tumorigenic potential of ESC seems to be reduced when cell is pre-differentiated in vitro [158, 159].

Neural stem cells (NSC) can improve motoric recovery and cognition [160–162].

Bone-marrow-derived stem cells (BMSC) showed a significantly improved functional outcome (as measured by the results of the rotarod test and the modified neurological severity score) in rats [161]. BMSC have been proven migrate to the injured brain and to improve cognitive dysfunction related to TAI, when transplanted 10 days after trauma [163]. BMSC survived after both intra-arterial and intravenous administration, after which cells migrated to the site of injury expressing neural cell markers [164, 165].

It is not entirely clear whether the beneficial effect of stem cell transplant is caused by either direct cell replacement, neurotrophic factors of bio-bridging. It has been shown that neurotrophic factors are released after stem cell transplant, leading to the conclusion that they form a key role in neuronal regeneration after stem cell implant [157, 160, 162].

Stem cell therapy has only been tested in vitro and in animal studies. No inpatient studies with stem cells have been performed so far. Since stem cell therapy focuses on neuronal regeneration and repair, it is probably more valuable in the chronic reparative phase and not in the acute stage of ICU management. Solid evidence on the exact timing and effectiveness of stem cell therapy is not yet available.

Neuronal axons of the central nervous system lack regenerative capacities, caused by a variety of pathways. Multiple agents addressing these pathways have been investigated, showing possible improvement of axonal outgrow and functional recovery in preclinical studies [166, 167].

Other studies focus on components the axonal membrane and support membrane repair [43, 168–170].

Despite a wealth of research into targeted treatments for TAI, a more bench-to-bedside approach is needed before we expect to have a breakthrough in this respect. A possible explanation for the lack of human treatment, despite the wealth of animal studies, is the incongruence in the pathological timelines between humans and rodents. There appears to be no suitable conversion rate between the two [171]. This makes the translation from experimental studies into clinical practice difficult.

### Most important findings

In order to make a clear distinction between diffuse axonal injury by non-traumatic causes and diffuse axonal injury due to trauma, we suggest that the term traumatic axonal injury, or TAI, should be used. The term DAI should be abandoned when speaking purely about the traumatic version.

Direct shear injury and tearing of axonal fibers seems to be of less clinical importance than the secondary axotomy, caused by a cascade of molecular pathways. Primary damage is a result of more massive injury and therefore probably does not exist in all TAI patients. Besides this, primary damage already occurs prior to arrival at the hospital and therefore leaves little to no options for treatment. Secondary axotomy is a slower process, and therefore leaves a window of opportunity for treatment.

**Fig. 3:**
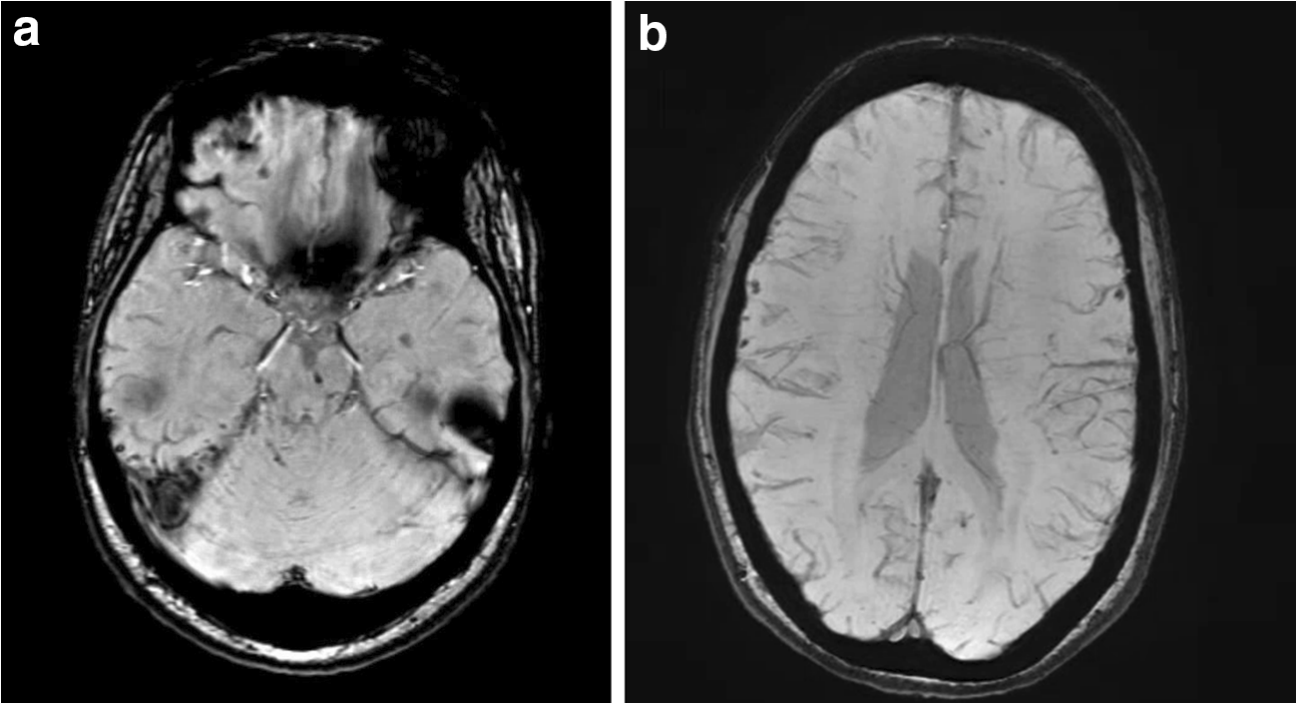
Susceptibility-weighted imaging (SWI) MRI series of a young patient with TAI. Note the susceptibility artifacts in the brain stem, temporal lobes, and cerebellum (**a**) and splenium of the corpus callosum (**b**). Despite grade 3 TAI, the patient was awake and communicating

Histopathological assessment of TAI injury has more historic rather than clinical relevance. Imaging, however, is of great clinical importance. MRI, and various on MRI-based techniques, seem to have the highest capability of identifying TAI lesions (Fig. 3). However, logistic concerns and safety during transportation to and from the ICU makes most polytrauma and severe TBI-patients are only eligible for CT-scanning. Evolution of MRI techniques will likely provide a breakthrough in this respect in the future.

Ideally, there should be a biomarker to identify TAI and to predict outcome. Currently, there are several potential biomarkers being researched. However, most of these biomarkers seem to be nonspecific for TAI, since they are present in all types of severe TBI. A biomarker specific for TAI has yet to be identified, but is a highly important area of future research, including research in the field of proteomics.

Currently, treatment enhancing regeneration of neural cells is not available. Some fundamental in vitro research on stem cell therapy seems promising in experimental studies. No studies have shown effectiveness of this therapy yet. The potential tumorigenic potential of stem cells calls for great caution with this technique into practice [158, 159]. Since the effect of stem cell transplantation might be caused by neurotropic factors, administration of these factors alone might be a promising field of research.

Treatment preventing secondary axotomy seems to be most promising. Avoiding axonal damage is better than repairing it. A plethora of experimental research is directed at finding a possible treatment in the window of opportunity between the primary and secondary axotomy phases. A bench-to-bedside approach from centers with broad experience in the treatment of TBI is required.

In daily practice, a cumbersome factor in both research and clinical decision-making for patients with TAI is the unpredictability of the outcome. Since many of the clinical decisions and informed consent talks are based on outcome data from the scientific literature, it is essential to commit further scientific efforts to report long-term longitudinal data of TAI patients, both isolated and within large severe TBI cohorts. Evidence in TBI, despite massive research efforts, remains weak [172]. Together with fundamental research efforts, this will bring new insights into the potential treatment optimization and future possibilities for TAI patients.

#### Clinical highlights and key points

- Primary axotomy is a mechanical lesion of the axon which occurs at the moment of trauma, followed by a “secondary” axotomy, involving apoptotic and degenerative processes (hydroxylation of proteins), especially at the level of the microtubules.
- Both primary and secondary axotomy may be the cause of coma despite the absence of increased intracranial pressure, depending on the foci of injury (e.g., bilateral thalamic and brainstem reticular formation).
- The Adams classification has never been validated in MRI studies. “Grade III” TAI, seen on imaging studies and sometimes used as a marker of the severity of the lesions, has no proven association with outcomes.
- TAI should be treated the same way as severe TBI, according to the guidelines, but centers with interest in TBI research should be aware of bench-to-bedside research efforts and participate in clinical trials for promising compounds from basic science research.
- As with other investigated therapies, timing is essential in TBI. The window of opportunity between primary and secondary axotomy is short.

## Conclusion

The prognostic value of TAI lesions seen on susceptibility-weighted MRI series has not yet been elucidated. Whether TAI requires targeted treatment different from regular severe TBI ICP-targeted therapy is still unclear. The chain of events involved in the pathophysiology further research in order to improve patient outcomes and potentially discover targeted therapies.
